# A Note on an Exon-Based Strategy to Identify Differentially Expressed Genes in RNA-Seq Experiments

**DOI:** 10.1371/journal.pone.0115964

**Published:** 2014-12-26

**Authors:** Asta Laiho, Laura L. Elo

**Affiliations:** 1 Turku Centre for Biotechnology, University of Turku and Åbo Akademi University, Turku, Finland; 2 Department of Mathematics and Statistics, University of Turku, Turku, Finland; Oklahoma State University, United States of America

## Abstract

RNA-sequencing (RNA-seq) has rapidly become the method of choice in many genome-wide transcriptomic studies. To meet the high expectations posed by this technology, powerful computational techniques are needed to translate the measurements into biological and biomedical understanding. A number of statistical procedures have already been developed to identify differentially expressed genes between distinct sample groups. With these methods statistical testing is typically performed after the data has been summarized at the gene level. As an alternative strategy, developed with the aim to improve the results, we demonstrate a method in which statistical testing at the exon level is performed prior to the summary of the results at the gene level. Using publicly available RNA-seq datasets as case studies, we illustrate how this exon-based strategy can improve the performance of the widely used differential expression software packages as compared to the conventional gene-based strategy. In particular, we show how it enables robust detection of moderate but systematic changes that are missed when relying on single gene-level summary counts only.

## Introduction

Deep sequencing of RNA (RNA-seq) has rapidly become a widely used technique to characterize transcriptomes. Whilst facilitating detailed mapping of the transcriptome over different cell types, perturbations and states, and providing superior sensitivity over expression microarrays, the method has generated high expectations [1]–[3]. To fully realize its potential, effective computational methods are needed in the analysis of the RNA-seq datasets [1], [4].

A fundamental research aim in RNA-seq studies is the identification of differentially expressed genes between distinct sample groups (e.g., healthy and disease). Accordingly, a number of statistical tools have already been developed for this task, including methods based on negative binomial models [5]–[8], non-parametric approaches [9], [10], and transformations of the read counts for linear modelling [11], [12]. Currently, however, there is no clear consensus on the best practices to detect differential expression from RNA-seq data [13], [14] whilst the field continues to develop.

Currently, the most common strategy for statistical analysis of RNA-seq data is based on the use of gene-level read counts [1], [13], [15], [16], which can be obtained, for instance, by mapping the sequenced reads to defined genes in Ensembl [17] or RefSeq [18]. Other approaches have also been developed for assembling the gene and transcript models from the data and calculating abundance estimates based on these models but this still remains a challenging task [8], [19]. In addition to detecting differential expression at the gene or transcript level, RNA-seq data can also be analysed for differential expression of isoforms based on exon-level expression signals [19]–[22]. While isoform analysis is not the goal of our approach, we present a method of gene-level differential expression analysis based on the direct analysis of the exon expression signals. Here, instead of summarizing the read counts across the exons prior to statistical testing, as is commonly done (typically using the total read count), we demonstrate the utility of an alternative strategy where the gene-level statistic is based on the statistical testing of the exon-level read counts. This is motivated by previous observations with Affymetrix gene expression microarrays indicating that statistical testing of probe-level expression signals, rather than gene-level summary values, can markedly improve the detection of differential gene expression, especially with small sample sizes [23]–[26].

**Figure 1:**
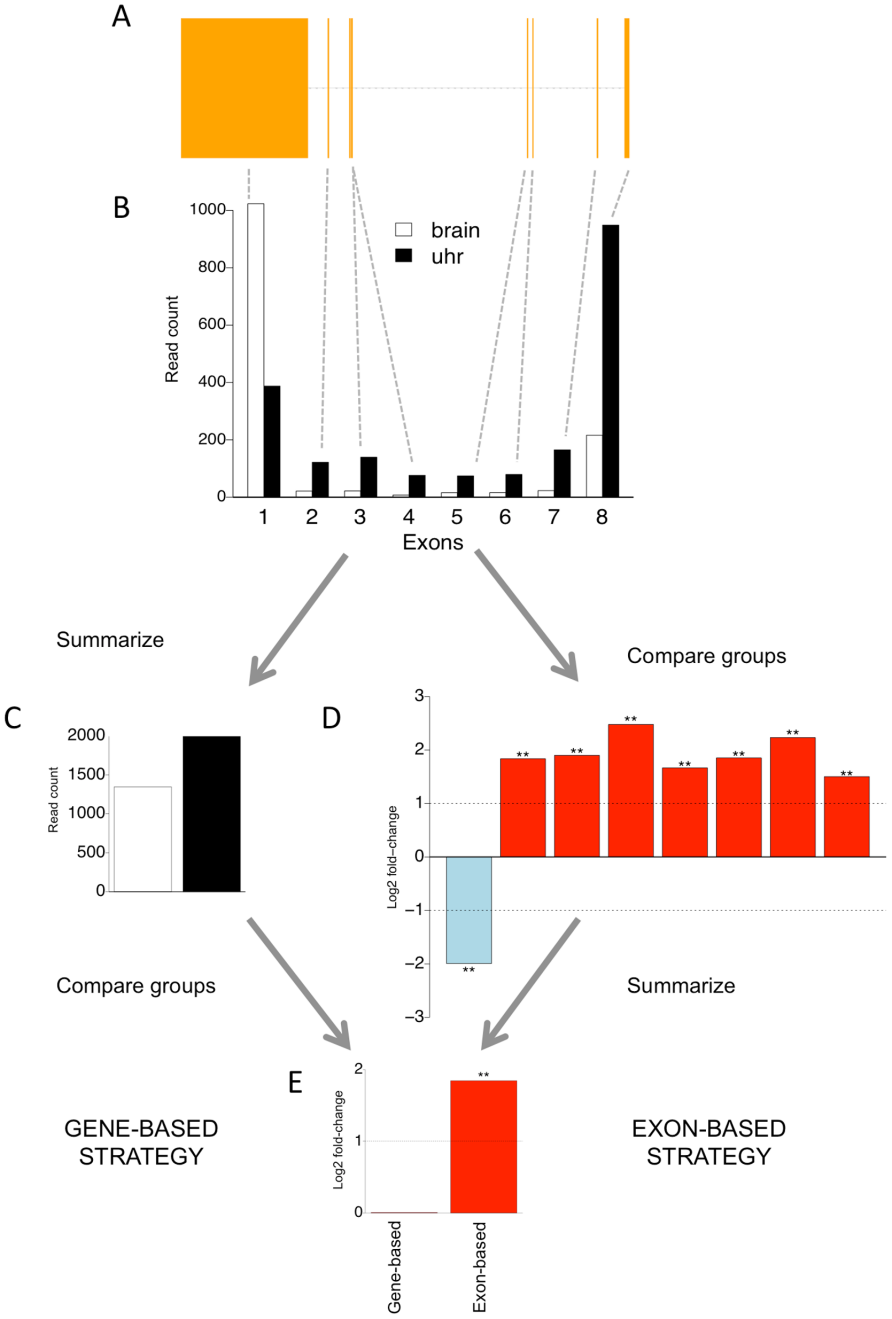
Schematic illustration of two alternative strategies (gene-based and exon-based) for detecting differential expression between two sample groups. The RNA-seq data are from the MAQC dataset, containing two types of biological samples: human brain reference (brain) and human universal reference RNA (uhr). (**A**) Exon structure of the gene DCUN1D5 (**B**) Separate read counts for the eight exons of the gene. (**C**) Normalized total read counts across all the exons for the gene. (**D**) Logarithmic (base 2) fold change between the sample groups separately for each exon. The number of stars above a bar indicates whether one or both of the two software packages (limma, edgeR) identify the particular exon as significant at *p*<0.05. (**E**) Gene-level log fold change between the sample groups obtained using directly the gene-level read counts (gene-based strategy; left bar) or by taking the median over the exon-level changes (exon-based strategy; right bar). The exon-based strategy supports differential expression (median *p* = 3.69e–06 and 1.64e–09 with limma and edgeR, respectively), whereas the conventional gene-based strategy suggests that the gene is equally expressed in both groups (*p* = 0.91 with both limma and edgeR). The fold changes were determined here using the limma software package.

Fig. 1 illustrates the benefit of the proposed exon-based strategy over the conventional gene-based strategy when detecting differential gene-level expression; a systematic significant change across most exons of the DCUN1D5 gene is lost with the gene-based approach mainly due to single exons, while the exon-based strategy identifies the gene as differentially expressed. This is because the gene-based approach is sensitive to extreme outliers, which makes the fold-change to shift to the direction of the extremely behaving exons. Due to the important role of alternative splicing in creating complexity, it is beneficial for a testing approach to be robust against single extreme values.

To systematically investigate the benefits of the proposed exon-based strategy in detecting differentially expressed genes, we consider two widely-used software packages that are conventionally applied to gene-level read counts, edgeR [7] and limma [11] and were also found to perform highly competitively in recent comparison studies [12], [14], [16]. However, our testing approach can be combined with any method working on gene- or transcript-level read count values. For the analyses presented, we have used two publicly available RNA-seq datasets as case studies. In the first case study, we demonstrate how our exon-based strategy can improve the sensitivity and specificity of the detections as compared to the traditional gene-based strategy in the MicroArray Quality Control (MAQC) benchmark data [27], [28]. In the second example study, we demonstrate the utility of the exon-based strategy in a more challenging real dataset involving substantial heterogeneity between individuals [29].

## Methods

### Exon-based strategy

A schematic illustration of the exon-based strategy is shown in Fig. 1. The underlying idea is to perform statistical testing (with e.g., limma or edgeR or any statistical testing approach suitable for RNA-seq data) separately for each exon and then aggregate the results at the gene level. In the present study, we defined the gene-level score as the median of its exon-level significance *p*-values, taking into account the directions of the changes. More specifically, if we denote by *x_i_* the estimated log2 fold change of an exon *i* and by *p_i_* the corresponding *p*-value obtained from the statistical testing, we determined the median over the signed log-transformed *p*-values 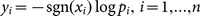, where *n* is the number of exons in the particular gene and *sgn* is the sign function. The log transformation forces the least significant *p*-value to zero. Alternatively to median, the method can, in principle, be used with any mean descriptor preferred.

For determining the exon-level *p*-values, we considered two popular R/Bioconductor packages for detecting differential expression in RNA-seq data, limma [11] and edgeR [7], and applied them to both gene- and exon-level count data using the default settings and following the instructions described in the package manuals. Statistical significance of a median *p*-value score was assessed by comparing the observed value to the null distribution obtained under the assumption that the exon-level *p*-values were uniformly distributed, taking into account the number of exons per gene. False discovery rates (FDR) were determined using the Benjamini-Hochberg multiple testing adjustment method [30]. With the exon-based strategy, we additionally ensured that the median *p*-value was below the corresponding FDR level by considering the maximum of these two values when ranking the genes or calling them differentially expressed.

Prior to the analysis, we filtered out very lowly expressed exons on the basis of their overall average count across the biological conditions as recommended for example in [31]. For a gene to be included in the analysis, we required that at least two of its exons had an overall average above one. Single exon genes were omitted here, as their result would not differ from that of the gene-based approach.

### Datasets

#### MAQC benchmark data

The MAQC RNA-seq data were downloaded from the Sequence Read Archive (SRA accession SRA010153). The data contain two types of biological samples: human brain reference (brain) and human universal reference RNA (uhr), both of which have been assayed using seven lanes on the Illumina Genome Analyzer II sequencing platform [27]. The reads were aligned to the human genome (hg19) using Tophat (version 2.0.4), splitting the reads into at least 17 bp segments and allowing up to one mismatch per segment. Gene- and exon-level read counts were determined based on RefSeq annotations using the python scripts incorporated in the DEXSeq (version 1.4.0) R/Bioconductor package. The corresponding qRT-PCR data were downloaded from the Gene Expression Omnibus (GEO accession GSE5350) and processed similarly to Bullard et al. [27]. Briefly, genes were required to be classified as present in at least three out of the four replicate qRT-PCR measurements in at least one of the sample groups (brain or uhr). Genes with an absolute log fold change above a cutoff value in the qRT-PCR data were then considered as differentially expressed (gold-standard positives). Various cutoff values were used at increasing stringency between 0.5 and 5 with increments of 0.1, yielding 601 to 88 gold standard positive genes, respectively. The 101 genes with absolute log fold change below 0.2 were considered as equally expressed (gold-standard negatives).

#### RNA-seq data on unrelated Nigerian individuals

The RNA-seq data were downloaded from the European Nucleotide Archive under the accession number SRP001540. The samples have been assayed using the Illumina Genome Analyzer II platform at two different centres using different read lengths (46 bp or 35 bp). The reads were aligned to the human genome (hg19) using Tophat (version 1.4.0) and default settings. Gene- and exon-level read counts were determined based on RefSeq annotations using the python scripts incorporated in the DEXSeq R/Bioconductor package.

## Results and Discussion

### Case study 1: MAQC benchmark data

We first assessed the performance of the gene- and exon-based strategies in the MAQC RNA-seq data, generated for benchmarking purposes [27], [28]. A major benefit of the MAQC data is that there are quantitative real-time polymerase chain reaction (qRT-PCR) data available on hundreds of genes that can be used for the evaluation of the different approaches.

**Figure 2:**
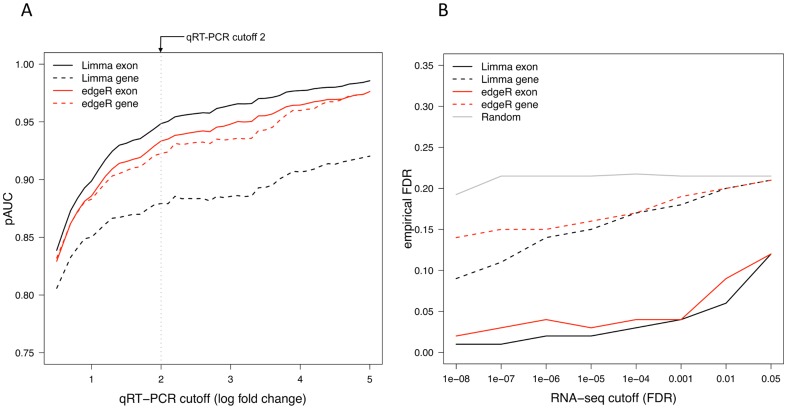
Comparison of the gene- and exon-based strategies in terms of qRT-PCR-derived gold standard in the MAQC data. Following the approach in [27], we considered a gene as a gold standard negative if its absolute log fold change in the qRT-PCR data was less than 0.2 and as a gold standard positive if its absolute log fold change in the qRT-PCR data was above a predefined cutoff value. (A) Partial area under the ROC curve (pAUC, *y*-axis) at various qRT-PCR cutoff values with increasing stringency were considered between 0.5 and 5 with increments of 0.1 (*x*-axis). At each cutoff, the performance of each method was assessed in terms of their receiver operating characteristic (ROC) curves and the corresponding partial areas under the curves (pAUC) at specificity of 0.8 (*y*-axis). (B) The empirical false discovery rate (empirical FDR, *y*-axis) as a function of different FDR cutoffs for the RNA-seq data (*x*-axis), using the qRT-PCR gold standard log fold change cutoff of 2 to determine the qRT-PCR gold standard positives. Genes with log fold change below 0.2 in the RNA-seq data (*x*-axis) were filtered out prior to determining the empirical FDR. To calculate the random curve, for each method and cutoff an equal number of genes was randomly selected to the result list and compared against the gold standard positive and gold standard negative gene lists. The results were then averaged across the randomizations.

Using the qRT-PCR data as a gold standard, we constructed receiver operating characteristic (ROC) curves for the different gene- and exon-based differential expression statistics. To summarize the performance in single values, we determined the partial area under the curves (pAUC) at a specificity of 0.8, standardized to have a maximal value of 1.0 (Fig. 2A). The pAUC was selected as the main evaluation criterion, since only identifications at low false positive rates are typically selected for further investigations in RNA-seq studies. Across the different cutoff values for the gold standard positives, the exon-based strategy systematically produced a higher pAUC-value than the corresponding gene-based strategy with both edgeR and limma. This demonstrated the improved sensitivity and specificity of the exon-based strategy compared to the conventional gene-based strategy. Not unexpectedly, increasing the fold change cutoff for the gold standard positives increased the ROC performance of both gene- and exon-based methods.

The qRT-PCR validations also enabled us to calculate the empirical false discovery rates of the detections (Fig. 2B). Across the different RNA-seq statistical testing FDR cutoffs, the exon-based strategy provided systematically lower empirical FDR values than the gene-based strategy for both limma and edgeR. This indicates that the FDR control of the exon-based strategy compares favourably to that of the gene-based strategy.

**Figure 3:**
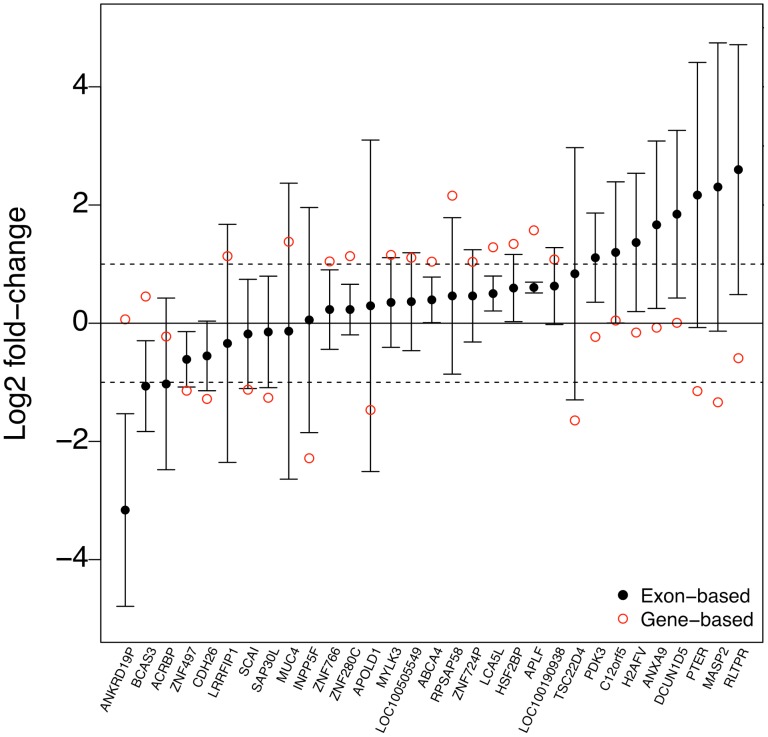
Robustness against single exons. Exon-wise and gene-based log fold changes for 31 genes in the MAQC data that showed largest differences between the gene- and exon-based strategies. The black dots and error bars show the median and standard deviation of the exon-level values, respectively; the red open circles are the corresponding gene-based values. We selected here genes with more than three exons, a fold change above 2 and *p*-value (or median *p*-value) below 0.05 with either of the strategies and fold change to the opposite direction or *p*-value difference above 0.1 with the other strategy, according to the values calculated with the limma software package. Among these genes, only MASP2 contained measurements also in the qRT-PCR data and they supported well the exon-based result (log2 fold-change of 1.85 in the qRT-PCR data), further confirming the utility of the exon-based strategy.

In order to take yet a closer look at the differences between the gene- and exon-based strategies, we examined the largest discrepancies between the detections in the MAQC data (Fig. 3). This suggested that the largest differences typically corresponded to genes for which only a single or relatively few exons behaved differently from the majority of exons, causing the gene-based estimate to deviate markedly from the median of the exon-level changes (see Fig. 1 for an example). This illustrated the robustness of the exon-based strategy against single deviant values, which can be expected to occur, for instance, due to alternative splicing events.

### Case study 2: RNA-seq data on unrelated Nigerian individuals

To assess the performance of the exon-based strategy in more complex real datasets involving substantial biological variation between replicates, we considered RNA-seq data on lymphoblastoid cell lines derived from unrelated Nigerian individuals as part of the International HapMap project [29]. For the present study, we compared the expression levels between 29 males and 29 females.

**Figure 4:**
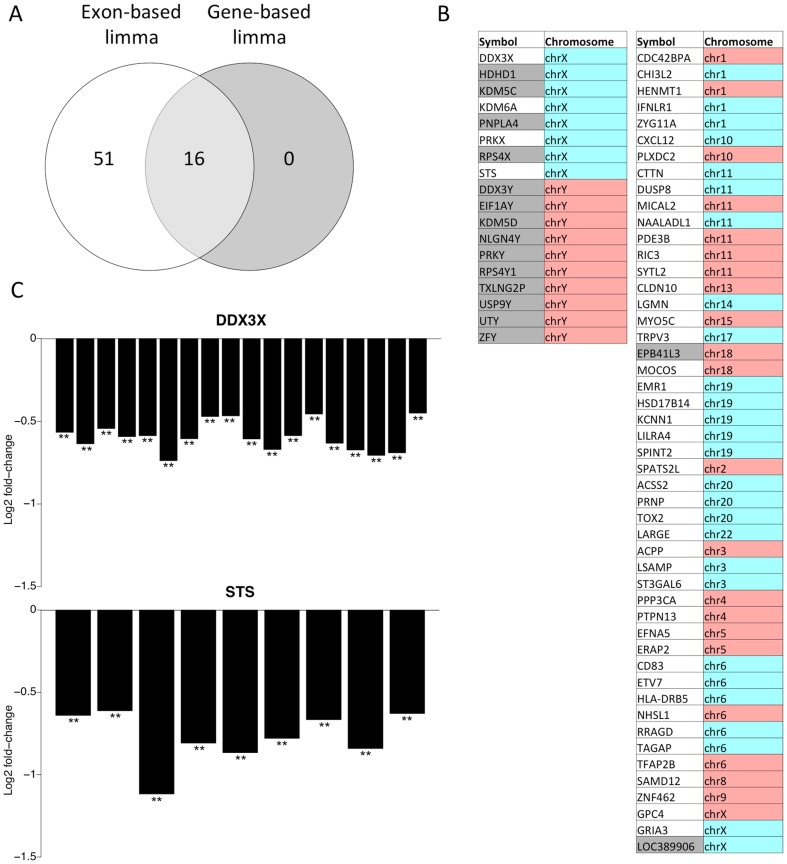
Differentially expressed genes between males and females in a population of Nigerian individuals. (**A**) Significant detections identified with gene-based or exon-based limma at FDR<0.05, an absolute fold change of at least 1.5 and at least 5 exons. With the exon-based strategy, we additionally required that the median *p*-value was below 0.05. Notably, all the gene-based detections were also found using the exon-based strategy, while the list of exon-based detections missed by the gene-based strategy contains 51 genes (**B**) The 67 genes detected using the exon-based approach with limma; genes reported as sex-specific in earlier studies or belonging to chromosome Y are shown on the left side. Genes detected also with the gene-based approach are highlighted with grey background. Red background in the chromosome column denotes higher expression in male than in female, blue vice versa. (**C**) Two examples of genes on the X chromosome that have previously been reported as sex-specific [32], [33] but were detected here only with the exon-based strategy. Both genes show moderate but systematic changes across the exons (*x*-axis). The number of stars above a bar indicates if only one or both of the two software packages (limma and edgeR) identify the particular exon as significant at *p*<0.05. The fold changes (female vs. male) were determined using the limma software package.

Focusing only on the most promising genes identified at FDR<0.05 and showing at least a 1.5-fold change between the male and female groups across at least five exons, we identified 16 and 67 genes using limma with the gene- and exon-based strategies, respectively (Fig. 4A and B). All of the gene-based detections were among the exon-based detections (highlighted in grey in Fig. 4B). In general, the genes missed by the gene-based strategy included genes with moderate but systematic changes across majority of their exons as well as some relatively low-abundance genes showing systematic changes across all exons. It is possible to detect the latter by the exon-level strategy due to the increased statistical power derived from having several measurements per gene. Fig. 4C illustrates two such example genes from the X chromosome that have previously been reported as sex-specific [32], [33] but were detected here only with the exon-based strategy. Finally, investigation of the differentially expressed genes identified by the exon-based limma analysis revealed their significant overlap with both Y and X chromosomes (*p*-values 3.29e–7 and 0.002, respectively; David tool [34]). A systematic comparison of the detections to the previously reported sex-specific genes on the X chromosome [32], [33] and genes on the Y chromosome also revealed a high overlap as shown in Fig. 4B (left panel). Of the common detections between the gene- and exon-based strategies, nearly 90% were among the genes listed in these publications. Four of the exon-based detections that were missed by the gene-based strategy were also among these previously reported sex-specific genes (DDX3X, KDM6A, PRKX and STS). The other genes detected by the exon-based strategy outside the X and Y chromosomes included, for instance, an enriched number of targets of the sex determining region Y (SRY) protein and the SRY-box 9 (SOX9) (*p*-values 0.004 and 0.002 respectively, David tool [34]). These observations illustrate the ability of the exon-based strategy to identify biologically relevant candidates, missed by the gene-based strategy, also in complex settings, which supports the high potential of the proposed strategy.

## Conclusions

Taken together, we demonstrated in this study how an exon-based strategy can significantly increase the sensitivity and specificity of the widely used differential expression methods for RNA-seq data over the conventional gene-based strategy. In particular, we observed that taking advantage of the exon-level signals enabled detection of such moderate but systematic gene expression changes that were missed by the gene-based strategy relying on single gene-level summary counts only. Additionally, our results showed how the gene-based approaches are prone to effects of single exons, while the exon-based strategy is robust against them.

Although we focused here on the most basic design of comparing two groups of samples, the exon-based strategy extends naturally to more complex study designs and to basically any current or future test statistic for detecting differential expression. It is not limited to a specific gene or transcript model either but can be applied to any user-defined feature model, such as windows across de novo assembled gene contigs.
